# Aircraft Aerodynamic Parameter Detection Using Micro Hot-Film Flow Sensor Array and BP Neural Network Identification

**DOI:** 10.3390/s120810920

**Published:** 2012-08-07

**Authors:** Ruiyi Que, Rong Zhu

**Affiliations:** State Key Laboratory of Precision Measurement Technology and Instruments, Department of Precision Instruments and Mechanology, Tsinghua University, Beijing 100084, China; E-Mail: katykob@163.com

**Keywords:** hot-film flow sensor, aerodynamic parameters, BP neural network

## Abstract

Air speed, angle of sideslip and angle of attack are fundamental aerodynamic parameters for controlling most aircraft. For small aircraft for which conventional detecting devices are too bulky and heavy to be utilized, a novel and practical methodology by which the aerodynamic parameters are inferred using a micro hot-film flow sensor array mounted on the surface of the wing is proposed. A back-propagation neural network is used to model the coupling relationship between readings of the sensor array and aerodynamic parameters. Two different sensor arrangements are tested in wind tunnel experiments and dependence of the system performance on the sensor arrangement is analyzed.

## 1. Introduction

Small unmanned air vehicles (UAVs) and micro air vehicles (MAVs) are attracting growing interest for their multi-purpose applications [1]. However, they suffer from certain particular characteristics such as small Reynolds number, low inertia, low flight speed, rolling instability and so on [2], which cause flight instability and make them highly prone to wind. To improve the performance of MAVs/UAVs, their aerodynamic parameters need to be introduced into flying control systems, as complementary information to inertial guiding systems and auto pilots. Conventional techniques for detecting these parameters, mostly based on Pitot tubes or electromechanical self-orienting vanes [3,4], usually protrude outside the aircraft [5], need hard mechanical ties to the body and/or intrusive pneumatic links inside and would be easily damaged while landing. What's more, installing more than one device, especially distributing sensors on flapping wings or flexible membrane wings for detecting multiple aerodynamic parameters, calls for more space, power and payload which can hardly be afforded by small aircrafts.

Redundant strip pressure sensors mounted on airfoil skins may be used to obtain aerodynamic parameters [5,6], however both the sensing technology and data processing methods of the methodology seem too complicated, and the angle of sideslip could not be detected. The method described in [7] required a hemispherical nose installed on the aircraft, which limited its application.

Aerodynamic parameters can be also inferred indirectly from the surface flow field around the wing using micro sensors [5,6]. The adoption of micro sensors has shown many technical and economical advantages compared with other conventional technologies. In the past two decades, several research groups have demonstrated micromachined flow sensors based on various principles, including thermal transfer [8,9], torque transfer, and pressure distribution.

**Figure 1.**
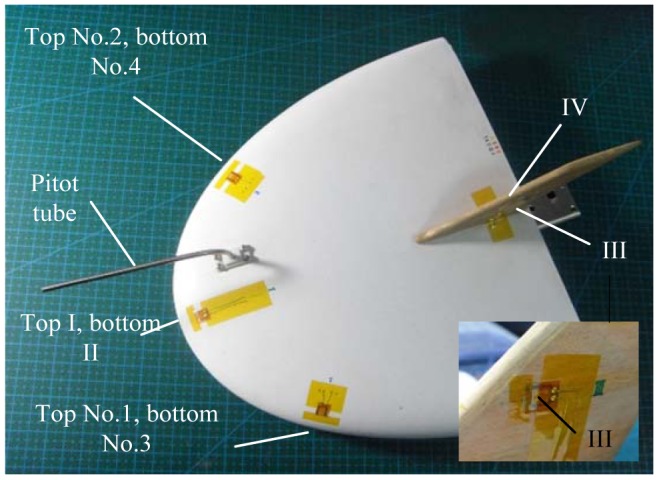
The collocation of hot film flow sensor array on a MAV.

In this paper, a novel and practical approach, by which the flow fields around the wing are measured and used to deduce multiple aerodynamic parameters of the aircraft, is proposed and validated through wind tunnel experiments. We propose the use of a micro hot-film flow sensor array and a back-propagation (BP) neural network to deduce three aerodynamic parameters: air speed, angle of attack and angle of sideslip. The sensors are tailor-developed on a flexible substrate and home fabricated. The hot film sensors are collocated on specific positions of the wing surface of a micro air vehicle, as illustrated in Figure 1. Two different arrangements: (1) hot film sensors 1, 2, 3, 4 and the Pitot tube; (2) hot film sensors I, II, III, IV and the Pitot tube, are proposed, tested and compared. The readings of the sensors are acquired and converted into digital signals as the inputs of the signal processor, whose outputs deduce the three aerodynamic parameters using a neural network-based data fusion technique. This methodology takes the merits of tiny sensing system, smooth and flexible traits, efficient data processing, and is thus especially suitable for applications on small UAVs and MAVs.

## 2. Operating Principles of Sensor System

The developed hot film sensor system has the features of flexible film, smooth surface, small size, low weight, and ease of mounting on versatile surfaces. The thin-film design makes it possible for detecting surface flow or sheer stress while guaranteeing the least disturbance of the surface flow.

### 2.1. Sensing Principles

The hot film flow sensor utilizes a thermal metallic thin-film deposited on a flexible substrate that is mounted on the surface of the aircraft wing. The thermal element serves as both Joule heater and temperature sensor. Under a constant bias power and zero flow rates, the thermal element achieves a steady-state temperature, which means the heat transfer system reaches equilibrium. If an external flow passes around the thermal element, the element experiences forced convective cooling. Accordingly, the temperature of the thermal element decreases, then the resistance of the element changes, and thus provides the information of the flow speed that governs the cooling rate.

### 2.2. Sensor Structure and Fabrication

**Figure 2.**
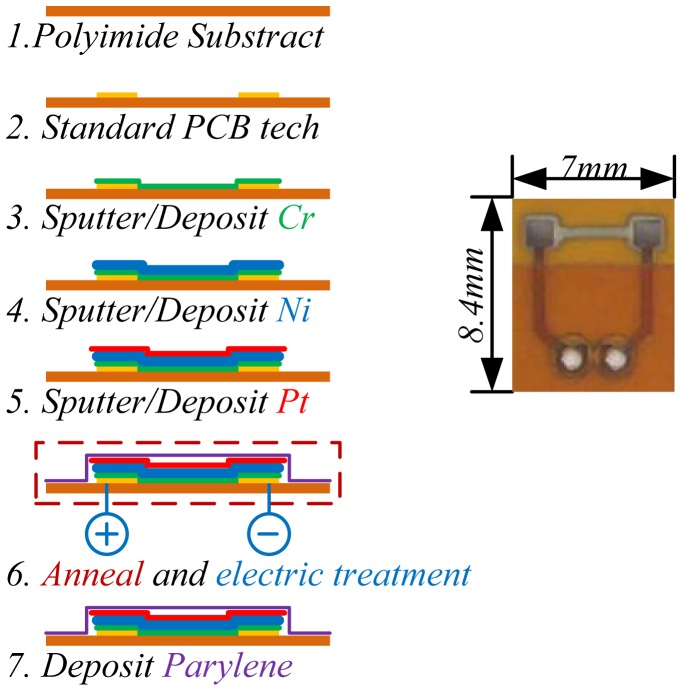
Diagram of the fabrication process of the hot film flow sensor.

The micro hot film sensors used in this work were homemade [10]. We used standard flexible PCB, *i.e.*, polyimide (PI) film, as the substrate of the sensor array and body of the electric wiring. PI has advantages of excellent thermal isolation and flexibility. Thermal element films (Cr/Ni/Pt) were sputtered on the substrate, and a parylene film was deposited on the sensor and served as encapsulation. The fabrication process is shown in Figure 2. During the fabrication and before parylene coating, the sensors were processed through an annealing treatment (160 °C for 3 h) and electric treatment (50 mW for 4 h) for aging. The temperature coefficients of resistance (TCR) of the fabricated hot film sensors were tested to be about 2,000 ppm/K, with linear coefficients higher than 0.99, and the resistances of the sensors were about 100 ohm.

### 2.3. Conditioning Circuit

**Figure 3.**
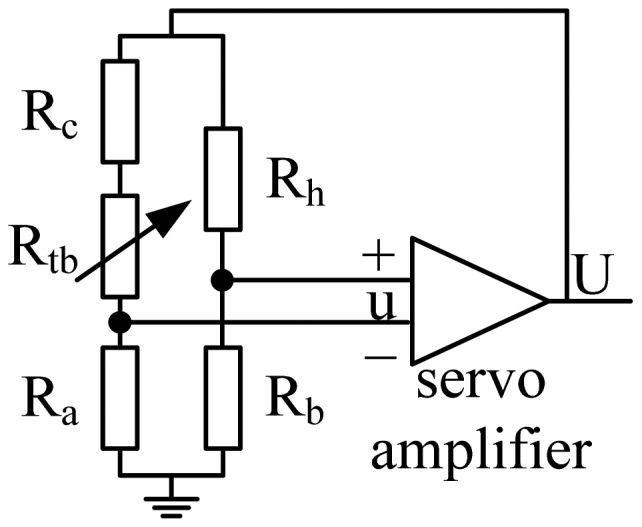
Scheme of a CTD mode driving circuit.

Hot film flow sensors can be operated either in constant voltage (CV), constant current (CC) and constant temperature difference (CTD) modes. In this work, the CTD mode, a scheme of which is shown in Figure 3, was used considering the superiorities of its sensitivity and dynamic response [11–13]. In the figure R_h_ is a thermal flow sensor, R_c_ is a temperature compensating sensor, R_tb_ is used to adjust the Joule heating level of R_h_ [14], R_a_ and R_b_ are the rest legs of the bridge.

**Figure 4.**
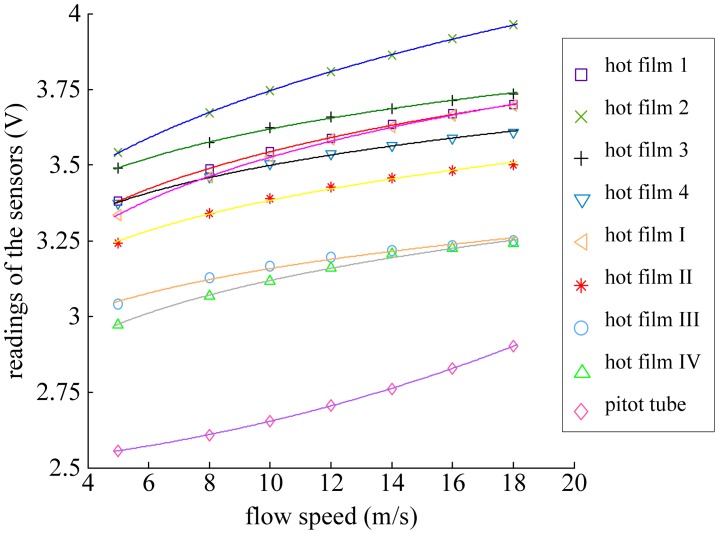
Monotone relationships between sensor readings and flow speed.

In CTD mode, a feedback is employed to maintain a constant temperature difference for the thermal flow sensor related to the ambient temperature except for very high frequency fluctuations [12–14]. Feeding U back to the top of the bridge restores the flow sensor's resistance to the original value via adjusting the Joule heating. Under the CTD mode, single sensor has a monotone relationship with the flow speed. The sensors' readings (including hot film sensors 1, 2, 3, 4, hot film sensors I, II, III, IV, and the Pitot tube) versus flow speeds tested in a wind tunnel are shown in Figure 4, where the flow speeds were set from 5 to 18 m/s (about 2 m/s per step). The results of Figure 4 reveal that the Pitot tube connecting with a pressure sensor had lower sensitivity at low airspeed and becomes more sensitive when the flow speed increases, while the hot film flow sensor exhibited the opposite tendency.

Connecting the flow sensors into the CTD circuit, the time constants of the sensor systems could be optimized to be less than 10 ms by setting the ratio R_a_/R_b_ as 10 and R_h_/R_b_ at about 1. In the calibration measurements, the sensitivity of the hot film flow sensors were estimated to be around 4 V^2^/(m/s)^n^ at the overheat ratio of 10% based on the sensor model *U^2^* = *A* + *B*·*V^n^* [10], where *U* is the reading of the sensor, *V* is the flow speed, *A*, *B* and *n* are the parameters determined through experimental calibration. In this study, *n* was tested to be about 0.2. The errors of flow measurements were less than 1 m/s. The resolution of the sensors reached 0.1 m/s, and the power consumption was tested to be about 20 mW at an overheat ratio of 10%.

## 3. Modeling of BP Neural Network

### 3.1. Basic Principle

**Figure 5.**
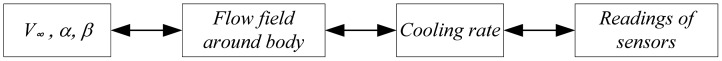
Relationship between the sensors' readings and aerodynamic parameters.

For a certain aircraft, the flow field around the wing surface depends on the free stream air speed *V*_∞_, the angle of attack *α* and the angle of sideslip *β* [8,9,14–17]. Therefore it is reasonable to detect these flight parameters by measuring the flow field around the wing surface. The flow field information around the wing contains two main components: the flow speed and the wall shear stress. Although, the both information is useful for inferring the flight parameters, we paid more attention to the flow speed in this study considering the low Prandtl number of about 0.7∼0.8 for air, which is the ratio of velocity boundary layer to thermal boundary layer. For an aircraft with a certain profile, the flow speed of the surface flow at several specific points on the wing surface can characterize the flow field around the wing, and thus can be used to deduce the aerodynamic parameters of the aircraft. The sheer stresses were detected using hot film flow sensors. Figure 5 depicts the relationship between the sensor readings and the three aerodynamic parameters.

It is generally impossible and unnecessary to measure all points on the surface, so we need to find out an appropriate sensor arrangement using as few sensors as possible to detect the aerodynamic parameters with least power and load. The work by Lian *et al.* [2] indicated that on the front edge of the wing, flow separation and vortexes seldom occurred, in addition, the flow field there changed violently with the change of air speed and flight aerodynamic angles, all of these implied that it was a good choice to mount the sensors on the front edge of the wing. In our work, two sensor arrangements shown in Figure 1 were employed. In the arrangement (1), hot film sensors 1, 2, 3 and 4 were mounted symmetrically on the top left, top right, bottom left and bottom right of the front edge of the wing, respectively. In the arrangement (2), hot film sensors I, II, III and IV were mounted on the top center, bottom center of the front edge, and the left side, right side of the vertical tail, respectively. Besides the hot film sensors, we also introduced a Pitot tube connecting with a pressure sensor in the system for helping to detect the airspeed.

### 3.2. Configuration of the System

The readings of five sensors (four hot film sensors and a Pitot tube) denoted as [*V*_1_, *V*_2_, *V*_3_, *V*_4_, *V_p_*] were acquired, which were used for deducing the three flight parameters: air speed *V*_∞_, angle of attack *α* and angle of sideslip *β*. Readings of the other sensor arrangement is denoted as [*V*_I_, *V*_II_, *V*_III_, *V*_IV_, *V_p_*]. The relationship between the sensors' readings and flight parameters can be formulated by:

(1) $$\left[V_{1},V_{2},V_{3},V_{4},V_{P}]T=f_{1}(V_{\infty },\alpha ,\beta \right)$$

(2) $$\left[V_{I},V_{\text{II}},V_{\text{III}},V_{\text{IV}},V_{P}]T=f_{2}(V_{\infty },\alpha ,\beta \right)$$

**Figure 6.**
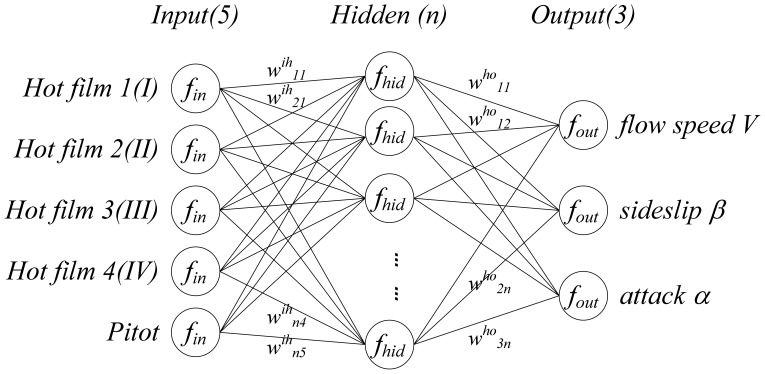
Structure of the neural network.

Intuitively, the relationship between the sensors' readings and the three flight parameters is a multiple input multiple output (MIMO) coupling system. As a matter of fact, *f* is generally a complex multivariate and nonlinear function, which plays a crucial role in the detection approach. An approximation of *f* can be obtained by various means, such as an analytical method using intrinsic fluid dynamic models, simulation, experiment-based identification or a mixture of the above. Theoretically, the analytical method is too complicated and inefficient (different airfoil results in different *f*); simulation is feasible in some sense, but is generally less valid; in contrast, the experimental identification is more reliable and effective. In this paper, we adopted experiment-based model identification method to develop *f*, by using a 3-layers BP neural network shown in Figure 6 as the model structure considering that it had been theoretically proved three layers of neural network could solve arbitrarily complicated nonlinear mapping problems [18]. For the application on small UAVs and MAVs, which requires both accuracy and simplicity, a 3-layer BP neural network with 9 neurons in the hidden layer was used, where the number of hidden neurons was determined through experimental testing. In Figure 6, normalization function *f_in_*, denormalization function *f_out_*, sigmoid function of the hidden layer *f_hid_* and transfer matrix *w^ih^*, *w^ho^* constitute the model structure of the function *f*.

## 4. Wind Tunnel Experiments and Analysis

### 4.1. Experimental Setup

**Figure 7.**
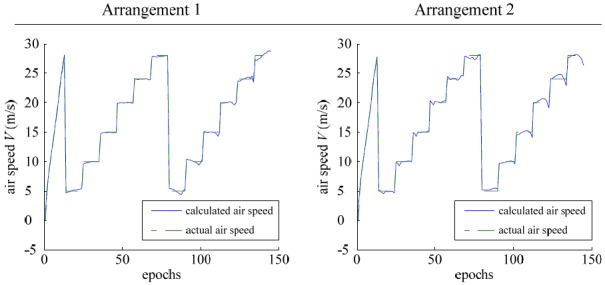
Setup of wind tunnel experiment.

The experiments were performed in a wind tunnel, as shown in Figure 7. The definition of the aerodynamic parameters, airspeeds *V*_∞_, angles of attack *α* and angles of sideslip *β*, are also shown.

In the experiment, a MAV with two sets of sensor arrangements mounted in the way described above was used, and the outputs of the sensors at different airspeeds *V*_∞_, angles of attack *α* and angles of sideslip *β* in the ranges of 0∼28 m/s (4∼5 m/s per step), 0°∼20° (2° per step) and 0°∼20° (2° per step) respectively were measured and recorded using a data acquisition unit.

The parameters of the BP network were determined through training, in which the output readings of the sensors were used as input samples of the network while the corresponding data of the actual flight parameters were used as the output samples of the network. The training rates were set various from 0.01 to 0.001 depending on the training accuracy. The hot film sensors were operated in the CTD modes and a temperature compensation approach [14] was used.

### 4.2. Experimental Results and Discuss

**Figure 8.**
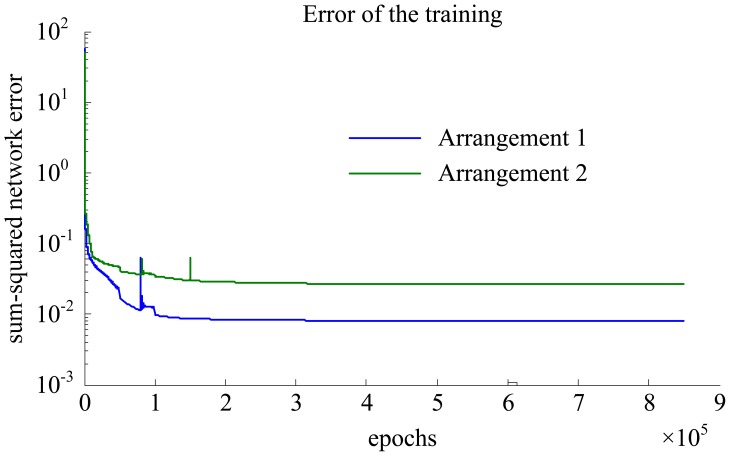
Neural network training results.

Data samples acquired in the experiments were used to carry out the model training. After 850,000 training epochs, the sum-squared network error reached less than 0.01 for the sensor arrangement 1 and less than 0.05 for the sensor arrangement 2, as shown in Figure 8.

**Figure 9.**
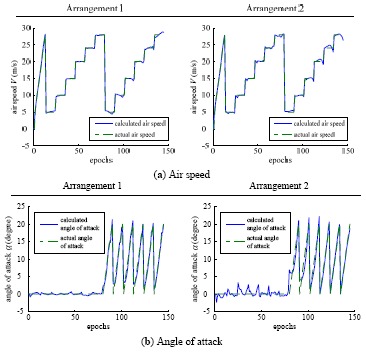

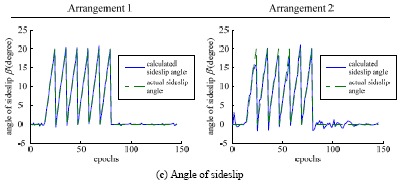
Comparative results of aerodynamic parameters.

The performances of the trained neural networks were evaluated by comparing the actual flight parameters with the model-based flight parameters calculated from the sensors. The comparative results are shown in Figure 9, where the blue solid lines represent the calculated flight parameters by using the network model with the sensor measurements as its inputs, and the green dashed lines represent the actual flight parameters correspondingly. The detailed test processes shown in Figure 9 are: firstly, the airspeed was set from 0 to 28 m/s (4∼5 m/s per step) while the angle of attack and the angle of sideslip were zero; afterwards, the angle of sideslip was set from 0° to 20° (2° per step) at different flow airspeed varied from 5 to 28 m/s (5 m/s per step) while the angle of attack kept at zero; finally, the angle of attack was set from 0° to 20° (2° per step) at different airspeed varied from 5 to 28 m/s (5 m/s per step) while the angle of sideslip kept at zero.

From the results in Figure 9, it can be seen that the calculated flight parameters by the sensor measurements fit the actual flight parameters very well. The measurement errors of air speed, angle of attack and angle of sideslip were approximately 0.27 m/s, 0.87°, 0.27° for the sensor arrangement 1, and 0.37 m/s, 1.44°, 0.93° for the arrangement 2. The measurement errors of the arrangement 1 were smaller than that of the arrangement 2 because the flow field around the front edge of the wing was much more stable than that around the vertical tail.

## 5. Conclusion and Future Work

A novel and practical methodology for detecting aerodynamic flight parameters using a micro hot film flow sensor array and neural network-based data fusion technology is proposed in this paper. The hot film sensor array is tailor-designed for the surface flow detection using a flexible substrate and thin-film deposition, and the BP neural network is used for simplifying the data fusion rather than the complicated theoretical analysis on the aircraft aerodynamics. Wing tunnel experiments validate the effectiveness of the proposed methodology. Two allocation arrangements of sensor array are investigated and tested. The comparative results show the arrangement 1 by allocating the sensor array on the front edge of the wing surface is preferable to arrangement 2 due to its higher accuracy. Our future work will focus on enhancing the stability and reliability of the hot film flow sensors and further optimizing the allocation of the sensor array on the aircraft wing.
